# PROMOTING SUCCESSFUL CHRONIC DISEASE SELF-MANAGEMENT THROUGH COMMUNITY-BASED INTERVENTIONS

**DOI:** 10.1093/geroni/igz038.266

**Published:** 2019-11-08

**Authors:** Tiffany R Washington, Laura N Gitlin, Matthew L Smith

**Affiliations:** 1 University of Georgia, Athens, Georgia, United States; 2 Drexel University, Philadephia, Pennsylvania, United States; 3 Texas A&M University, College Station, Texas, United States

## Abstract

Persistent chronic conditions are among the top leading causes of death in the U.S. The majority of older adults live with two or more chronic conditions. When poorly managed, chronic conditions can result in negative psychosocial and health outcomes such as low quality of life, diminished mood, workforce withdrawal, high healthcare utilization, and disability. Fortunately, chronic disease self-management interventions show promise in reducing the negative impact of chronic conditions on health status. This symposium will highlight formative work and recent findings of community-based interventions that promote successful self-management. To start, presenter one will describe findings from four studies that constitute the formative work necessary to inform implementation of self-management interventions in community and healthcare settings. Next, presenter two will report findings from an effectiveness trial of a technology-based intervention to support hypertension self-management. Then, presenter three will describe findings on the feasibility of a Health Passport tool to promote self-health management among individuals with physical limitations. Finally, presenter four will describe factors affecting attendance among African Americans with arthritis who participated in a national dissemination of Chronic Disease Self-Management Education programs. Collectively, these presentations will provide practical evidence and science-based recommendations for ways to increase successful chronic disease self-management and ultimately improve population health among older adults.

